# Anthropological analyses of 30 insertion/deletion autosomal markers in five major ethnic groups of Pakistan

**DOI:** 10.1080/20961790.2021.1933366

**Published:** 2021-08-28

**Authors:** Muhammad Adnan Shan, Julie Mechlenborg, Rebecca Røgen, Claus Børsting, Niels Morling

**Affiliations:** a Section of Forensic Genetics, Department of Forensic Medicine, Faculty of Health and Medical Sciences, University of Copenhagen, Copenhagen, Denmark; b Centre for Applied Molecular Biology (CAMB), University of the Punjab, Lahore, Pakistan

**Keywords:** Forensic sciences, forensic genetics, Qiagen, investigator^®^ DIPplex kit, anthropology, population genetics, individual identification

## Abstract

We investigated the forensic efficacy of the 30 insertion/deletion (Indel) markers included in the Qiagen Investigator® DIPplex kit in 529 Pakistani individuals from five major subpopulations in Pakistan (Punjabi, Pashtun, Sindhi, Saraiki, and Baloch). In the Sindhi population, the distribution of HLD81 and HLD97 alleles deviated from Hardy-Weinberg equilibrium after Bonferroni correction. The combined match probability ranged from 2.0E-12 (Pashtun and Baloch) to 1.0E-12 (Sindhi), and the mean paternity exclusion power varied from 0.995 (Punjabi, Sindhi, and Saraiki) to 0.996 (Pashtun and Baloch). The high combined power of discrimination (0.999 999 999 999 97) and low combined match probability (1.7E-12) for all subpopulations studied support the utility of the 30 Indel markers for forensic identification in the studied subpopulations. The allele frequencies of the Indel markers in the Pakistani subpopulations were compared with those from 18 other populations. The results show that the populations clustered according to geography. The subpopulations investigated in this work showed a close genetic relationship with others from Pakistan, as well as with South Central Asian and Middle Eastern populations. The results suggest that the Investigator^®^ DIPplex kit can be useful as a supplementary tool for human identification in the five Pakistani subpopulations investigated in this study.

Supplemental data for this article is available online at https://doi.org/10.1080/20961790.2021.1933366 .

## Key points

The Investigator^®^ DIPplex kit is useful as a supplementary tool for human identification.

Reference data for the alleles of the loci in the Investigator^®^ DIPplex kit were established for the Pakistani subpopulations Punjabis, Pashtuns, Sindhis, Saraikis, and Baloch.

The Baloch were genetically slightly different from the other investigated Pakistani subpopulations.

## Introduction

Pakistan is the sixth most populous country in the world with more than 212 million individuals [1]. Pakistan is divided into four provinces (Punjab, Khyber Pakhtunkhwa, Balochistan, and Sindh) with several major and minor ethnic groups [2]. The Indus River System and many ancient civilizations as well as invasions and migrations have been important in shaping the ethnic and linguistic groups in Pakistan [3,4]. The social life of many of the groups is organized in clans [5]. The Punjabis is the largest subpopulation (44.7%) of Pakistan [6]. The Pashtuns live in the Khyber Pakhtunkhwa and Baluchistan provinces. The Pashtun subpopulation speaks the Pashto language and practice Pashtunwali, a set of customs and cultural values (What is Pashtunwali? Feb 27, 2012 posting by Alley J to EDUKASI Blog; unreferenced, available from: https://edukasipresenttime.blogspot.com/2012/02/is-pashtunwali.html). The Sindh province of Pakistan is the historical home of the Sindhi group [7]. Immigrants from Persia, Turkey, and Saudi Arabia have contributed to the genetic heterogeneity of the Sindhi subpopulation [8,9]. The Saraikis live in the southern part of Punjab. They constitute 10.5% of the Pakistani population and speak Saraiki [10]. The Baloch people live in Balochistan in the southwes­tern part of Pakistan. Geographically, it is the largest province in Pakistan [11].

This study aimed to describe the allele frequency distribution of 30 insertion-deletion (Indel) markers in five major ethnic groups from Pakistan and investigate the forensic genetic effectiveness for future application in forensic casework. Indel markers consist of either a deletion or an insertion of nucleotides [12,13]. Indels are distributed throughout the human genome, and their lengths vary from one base to up to millions of bases with 2 − 4 bp Indels being the most abundant ones [12]. In comparison with Short Tandem Repeats (STRs), Indels have lower mutation rates, short amplicon sizes, and length variation characteristics that make them suita­ble for highly degraded samples in forensic and kinship analysis [14,15].

## Materials and methods

### Samples

Buccal swabs were collected on FTA cards from 529 unrelated healthy Pakistani individuals after acquiring signed informed consent. The individuals belonged to five major Pakistani subpopulations: 106 Punjabis from northern Punjab, 107 Pashtuns from Khyber Pakhtunkhwa, 103 Saraikis from southern Punjab, 104 Sindhis from Sindh, and 109 Balochs from Baluchistan. The study was approved by the Review Board/Ethical Committee of the University of the Punjab, Pakistan (D/No. 019/DFEMS). All samples were anonymized. Genomic DNA was extracted as previously described [16]. Purified DNA was quantified using the Qubit™ dsDNA HS (High Sensitivity) Assay Kit and a Qubit^®^ 3.0 Fluorometer (Thermo Fisher Scientific, Waltham, MA, USA) according to the manufactu­rer’s recommendation.

### Indel typing

The amplification of DNA samples was performed using the Investigator DIPplex Kit (Qiagen, Hilden, Germany) that includes 30 bi-allelic autosomal Indels and amelogenin. PCR cycling was performed on a GeneAmp PCR System 9700 Thermal Cycler (Applied Biosystems, Foster City, CA, USA) following the manufacturer’s protocol. The PCR amplicons were separated by capillary electrophoresis using 3500xL Genetic Analyzer (Thermo Fisher Scientific) and the POP-4™ polymer (Thermo Fisher Scientific). Allele allocation was carried out with GeneMapper^®^ ID-X Software v1.4 (Thermo Fisher Scientific) using the allelic ladder and the set of bins and panels provided by the manufacturer of the kit. The DIPSorter software (Qiagen) was used for the interpretation of the results.

### Statistical analysis

Population statistics including allele frequencies, Hardy-Weinberg equilibrium (HWE), linkage disequilibrium (LD), pairwise *F_ST_* values, and diversity measures were calculated using the Arlequin v3.5 software [17]. HWE analysis was carried out using 1 000 000 Markov Chain Monte Carlo (MCMC) steps and 100 000 dememorization steps. LD analysis was performed with 10 000 permutations. Correction for multiple testing was done according to Bonferroni. The observed heterozygo­sity (Ho), expected heterozygosity (He), matching probability (MP), typical paternity index (TPI), power of discrimination (PD), and power of exclusion (PE) were calculated with Powerstats v1.2 [18]. The mean paternity exclusion power (MPE), combined matching probability (CMP) and combined paternity indices (duos and trios) were calculated with DNAVIEW™ version 28.103 [19]. To estimate the genetic relationship between the five studied subpopulations (Supplementary Table S1) and other populations, data of previously published populations were collected (Supplementary Table S2). The pairwise *F_ST_* values were calculated using the POPTREE2 software [20]. The pairwise *F_ST_* values were visualized in a multidimensional scaling (MDS) plot using the package MASS (version 7.3-51.1) [21] and RStudio [22].

## Results

The Indel frequencies, Ho, He, MP, TPI, PD, and PE in the subpopulations studied are shown in Supplementary Table S1. The frequencies of the deletions varied from 0.259 to 0.741 in the Punjabi population, 0.299 to 0.668 in the Pashtun, 0.223 to 0.738 in the Sindhi population, 0.197 to 0.721 in the Saraiki population and 0.266 to 0.706 in the Baloch population as shown in Supplementary Table S1.

The Ho ranged from 0.311 for the markers HLD81, HLD97 (Sindhi) and HLD83 (Punjabi) to 0.575 for HLD58 (Punjabi). The average gene diversity over loci for all the subpopulations ranged from 0.465 (Punjabi and Saraiki) to 0.475 (Pashtun). All the loci were in HWE after Bonferroni correction for multiple testing (*P* <0.0017), except for HLD81 and HLD 97 in the Sindhi popu­lation (Supplementary Table S1). Both loci showed lower Ho than expected. Statistically significant LD was detected among the Indels after Bonferroni correction for three marker pairs (HLD45/HLD56, HLD81/HLD84, and HLD84/HLD128) in the Pashtun, one marker pair (HLD58/HLD83) in the Sindhi, and one marker pair (HLD77/HLD128) in the Saraiki population.

The PD ranged from 0.513 to 0.694. HLD128 was the most discriminating locus (Pashtun). The PE varied from 0.052 to 0.272. HLD58 had the highest PE value in Punjabis. In the studied subpopulations, the CMP ranged from 2.0E-12 (Pashtun and Baloch) to 1.0E-12 (Sindhi), the MPE ranged from 0.995 (Punjabi, Sindhi, and Saraiki) to 0.996 (Pashtun and Baloch), and the combined power of discrimination (CPD) ranged from 0.999 999 999 999 90 (Baloch) to 0.999 999 999 999 98 (Pashtuns) (Table 1). The combined paternity indices for trios and duos ranged from 2 190 (Saraikis) to 2 300 (Pashtuns) and from 145 (Saraikis) to 155 (Pashtuns), respectively (Table 1). The observed *F_ST_* values were statistically significantly different between the Punjabi and Pashtun subpopulations, as well as between the Baloch and the remaining groups (*P* = 0.005).

**Table 1.** Parameters of genetic diversity and forensic efficiency for 30 Indels in five Pakistani subpopulations.

Population	Ho_a_	He_a_	MPE_a_	CMP	CPI (trio)	CPI (duo)		CPD
Punjabi	0.464	0.467	0.995	1.2E-12	2220	146		0.999 999 999 999 94
Pashtun	0.462	0.477	0.996	2.0E-12	2300	155		0.999 999 999 999 98
Sindhi	0.425	0.471	0.995	1.0E-12	2230	149		0.999 999 999 999 94
Saraiki	0.435	0.466	0.995	1.1E-12	2190	145		0.999 999 999 999 96
Baloch	0.456	0.473	0.996	2.0E-12	2270	151		0.999 999 999 999 90
All	0.450	0.492	0.996	1.7E-12	2270	152		0.999 999 999 999 97

Ho: observed heterozygosity; He: expected heterozygosity; MPE: mean paternity exclusion power; CMP: combined matching probability; CPI: combined paternity index; CPD: combined power of discrimination. ^a^mean values.

**Figure 1.**
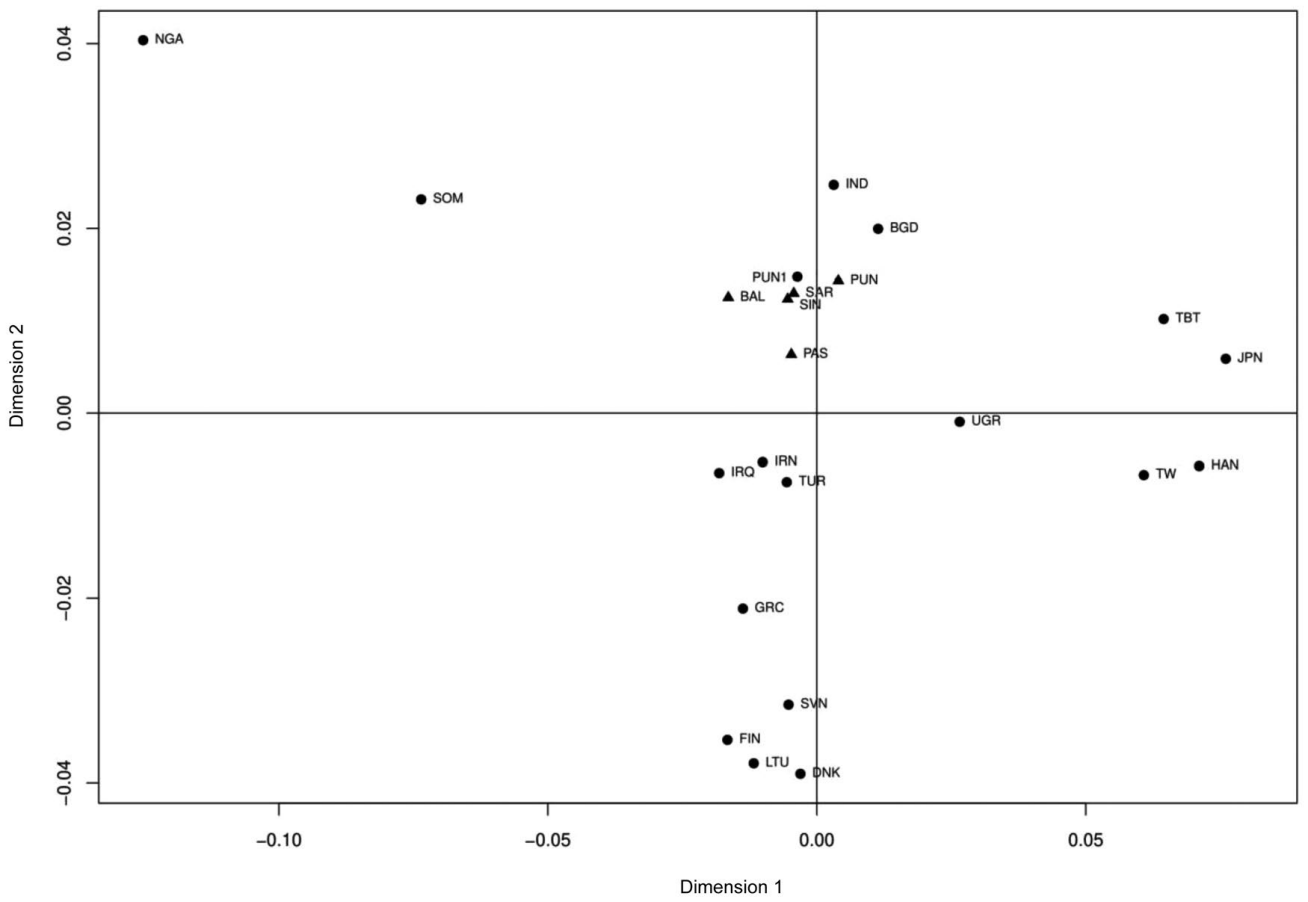
Multidimensional scaling (MDS) plot based on pairwise *F_ST_* values of five Pakistani subpopulations and 18 other populations. The studied Pakistani subpopulations indicated by triangles clustered closely together and to geographically neighbouring populations. BAL: Baloch (present study); BGD: Bangladeshi; DNK: Danish; FIN: Finnish; GRC: Greek; HAN: Han; IND: Indian; IRN: Iranian; IRQ: Iraqi; JPN: Japanese; LTU: Lithuanian; NGA: Nigerian; PAS: Pashtun (present study); PUN: Punjabi (present study); PUN1: Punjabi1; SAR: Saraiki (present study); SIN: Sindhi (present study); SVN: Slovenian; SOM: Somali; TUR: Turkish; TBT: Tibetan; TW: Taiwan people (the specific population is not available in original reference); UGR: Uyghur. The references to the original population data are shown in Supplementary Table S2.

To compare the genetic relationship among the Pakistani subpopulations, those from neighbouring territories, South Asia, East Asia, Middle East, Africa, and Europe, pairwise *F_ST_* genetic distances were calculated. Figure 1 shows an MDS plot of the data (stress level = 5.3%). The five subpopulations of this study clustered according to the geographic location and showed a close genetic relationship with those of other Pakistani, South Central Asian, and Middle Eastern populations.

## Discussion

This study demonstrated that the Investigator^®^ DIPplex kit is useful as a supplementary tool for human identification, especially in cases with genetic inconsistencies, and in supplementary testing in relationship testing, e.g., in cases with a few genetic inconsistencies in STR systems. The allelic data reported here can be used as population reference database for the studied subpopulations. The small degree of LD among some alleles at various loci is most probably due to non-random mating, as previously reported by Manzoor et al. [23].

Genetic differences among the investigated groups were small yet statistically significant among some groups. This study confirms the previous findings by Chishti et al. [24], who found some genetic dissimilarities between the Baloch and other Pakistani subpopulations.

It is important to carry out further studies to better understand the genetic structure of the Baloch subpopulation and its relation to the other subpopulations.

## Supplementary Material

Supplemental Material
