# Supplementary material for: Anthropological analyses of 30 insertion/deletion autosomal markers in five major ethnic groups of Pakistan
Source: Forensic Sci Res. 2021 Aug 28;7(2):202–6. doi: 10.1080/20961790.2021.1933366 (PMC9245971; doi:10.1080/20961790.2021.1933366)
Supplement: Supplemental Material [file TFSR_A_1933366_SM4019.zip › Supplementary-Material-S2 of 1933366.doc]

**Supplementary Table S2. The 23 populations included in the inter-population analysis of Pakistani subpopulations and other populations.**

| **Region** | **Population** | **Acronym** | **Number** | **Reference** |
| --- | --- | --- | --- | --- |
| **Pakistan** | Punjabi | PUN | 106 | This study |
|  | Pashtun | PAS | 107 | This study |
|  | Sindhi | SIN | 103 | This study |
|  | Saraiki | SAR | 104 | This study |
|  | Baloch | BAL | 109 | This study |
| **South Asia** | Punjabi, Pakistan | PUN1 | 260 | [1] |
|  | Bangladeshi | BGD | 132 | [2] |
|  | Indian | IND | 102 | [3] |
| **Middle East** | Irani | IRN | 253 | [4] |
|  | Iraqi | IRQ | 101 | [5] |
|  | Turkish | TUR | 93 | [5] |
| **East Asia** | Han | HAN | 129 | [6] |
|  | Tibetan | TBT | 136 | [6] |
|  | Uyghur | UGR | 123 | [6] |
|  | Japanese | JPN | 251 | [7] |
|  | Gaoshan | GS | 126 | [8] |
| **Europe** | Danish | DNK | 80 | [9] |
|  | Finland | FIN | 151 | [10] |
|  | Slovenian | SVN | 157 | [5] |
|  | Greek | GRC | 177 | [11] |
|  | Lithuania | LTU | 110 | [5] |
| **Africa** | Somali | SOM | 175 | [10] |
|  | Nigerian | NGA | 140 | [12] |

# The aconyms are explained in the legend to Figure 1.

# References

1. Shahzad M, Hussain M, Shafique M, et al. Population genetic data of 30 insertion–deletion markers in Punjabi population of Pakistan. Int J Legal Med. 2019;0551:511–512.
2. Al-Enizi M, Ge J, Ismael S, et al. Population genetic analyses of 15 STR loci from seven forensically-relevant populations residing in the state of Kuwait. Forensic Sci Int Genet. 2013;7:e106–e107.

3.	Hefke G, Davison S, D’Amato ME. Forensic performance of Investigator DIPplex indels genotyping kit in native, immigrant, and admixed populations in South Africa. Electrophoresis. 2015;36:3018–3025.

4.	Poulsen L, Farzad MS, Børsting C, et al. Population and forensic data for three sets of forensic genetic markers in four ethnic groups from Iran: Persians, Lurs, Kurds and Azeris. Forensic Sci Int Genet 2015;17:43–46.

5.	Tomas C, Poulsen L, Drobnič K, et al. Thirty autosomal insertion-deletion polymorphisms analyzed using the Investigator^®^ DIPplex Kit in populations from Iraq, Lithuania, Slovenia, and Turkey. Forensic Sci Int Genet. 2016;25:142–144.

6.	Bian Y, Zhang S, Zhou W, et al. Analysis of genetic admixture in Uyghur using the 26 Y-STR loci system. Sci Rep. 2016;6:19998.

7.	Nunotani M, Shiozaki T, Sato N, et al. Analysis of 30 insertion–deletion polymorphisms in the Japanese population using the Investigator DIPplex^®^ kit. Leg Med. 2015;17:467–470.

8.	Pepinski W, Abreu-Glowacka M, Koralewska-Kordel M, et al. Population genetics of 30 INDELs in populations of Poland and Taiwan. Mol Biol Rep. 2013;40:4333–4338.

9.	Friis SL, Børsting C, Rockenbauer E, et al. Typing of 30 insertion/deletions in Danes using the first commercial indel kit—Mentype^®^ DIPplex. Forensic Sci Int Genet. 2012;6:e72–e74.

10. Neuvonen AM, Palo JU, Hedman M, et al. Discrimination power of Investigator DIPplex loci in Finnish and Somali populations. Forensic Sci Int Genet. 2012;6:e99–e102.

11. Tomas C, Skitsa I, Steinmeier E, et al. Results for five sets of forensic genetic markers studied in a Greek population sample. Forensic Sci Int Genet. 2015;16:132–137.

12.	Du W, Peng Z, Feng C, et al. Forensic efficiency and genetic variation of 30 InDels in Vietnamese and Nigerian populations. Oncotarget. 2017;8: 88934–88940.

**Supplementary Table S3: Pairwise *F_ST_* values among five Pakistani subpopulations.**

| Populations | Baloch | Pashtun | Punjabi | Saraiki | Sindhi |
| --- | --- | --- | --- | --- | --- |
| Baloch | * | 0.0256 | **0.0000** | **0.0000** | 0.0053 |
| Pashtun | 0.0029 | * | 0.0113 | 0.0820 | 0.0897 |
| Punjabi | 0.0083 | 0.0034 | * | 0.2722 | 0.0140 |
| Saraiki | 0.0067 | 0.0021 | 0.0009 | * | 0.3500 |
| Sindhi | 0.0044 | 0.0022 | 0.0034 | 0.0008 | * |

The pairwise *F_ST_* values among the subpopulations are below the diagonal. The corresponding *P*-values are above the diagonal. Statistically significant values after Bonferoni correction for multiple testing are in bold.
